# Characteristics and outcome of pediatric and adult differentiated thyroid cancer with distant metastases

**DOI:** 10.3389/fendo.2026.1668565

**Published:** 2026-02-04

**Authors:** Ali S. Alzahrani, Lulu Alobaid, Eman Albasri, Afnan Hadadi, Abdulrhman Hakami, Fayha Abothenain, Deema Alturki, Najla Ewain, Ali Howaidi, Hindi Alhindi, Ghada Alskait, Yasser Aljufan, Shatha Alghaihb, Azzam Alkhalifah, Leenah AlAyoubi, Amani Abualnaja

**Affiliations:** 1 Department of Medicine, King Faisal Specialist Hospital and Research Centre, Riyadh, Saudi Arabia; 2 Department of Molecular Oncology, King Faisal Specialist Hospital and Research Centre, Riyadh, Saudi Arabia; 3 Department of Medicine, King Abdulaziz Medical City, Riyadh, Saudi Arabia; 4 Department of Pathology, King Fahad Medical City, Riyadh, Saudi Arabia; 5 Department of Pathology and Laboratory Medicine, King Faisal Specialist Hospital and Research Centre, Riyadh, Saudi Arabia

**Keywords:** adult thyroid cancer, differentiated thyroid cancer, distant metastasis, papillary thyroid cancer, pediatric thyroid cancer, thyroid cancer

## Abstract

**Introduction:**

Previous studies suggested differences in the histopathological and molecular characteristics and outcome between pediatric and adult differentiated thyroid cancer (DTC). In this study, we focused on the characteristics and outcome of distant metastases (DM) between pediatric and adult patients with DTC.

**Objectives:**

To compare DM between pediatric (pDTC) and adult DTC (aDTC).

**Patients and methods:**

We studied 35 pDTC (≤ 18 years) and 79 aDTC with DM seen over 20 years. The median age was 16 and 55 years and the F:M ratio was 3.4:1 and 1.7:1, respectively. Total thyroidectomy was performed in 91.4% and 93.7% and lymph node dissection in 94.3% and 64.6% of pediatric and adult patients, respectively. Radioactive iodine (RAI) ablation/therapy was administered to all patients except one.

**Results:**

pDM mostly involved a single organ (34/35), predominantly the lungs (32/35), and rarely the bone (2/35) and brain (1/35). By contrast, aDM were frequently multi-organ (45/79) involving lungs (72 patients), bone (50), brain (9), liver (10), kidneys (3), adrenal glands (2), breast (1) and soft tissues (3). Additional therapies (e.g. surgery, RAI) were administered to 28 and 78 pediatric and adult patients, respectively. At the last follow-up, only 1 (2.9%) vs. 6 (7.6%) patients had progressive metastases and 1 (2.9%) vs. 46 (58.2%) patients died due to DTC in pediatric and adult groups, respectively (P<0.0001). Other patients either were in an indeterminate status or had stable non-progressive metastases.

**Conclusions:**

pDM mostly involve single organs and have good outcome and rare mortality. aDM are frequently multi-organ, progressive and associated with high mortality of 58% over about 4.5 years.

## Introduction

Differentiated thyroid cancer (DTC) is the most common endocrine malignancy (1, 2). Its incidence has been increasing over the past 40 years although recent studies suggest more stable rates or even some decline in the incidence over the last few years (3, 4). This increasing incidence of DTC is not limited to adults, but pediatric DTC has also been increasing, at least in the last 2 decades (5–7). However, pediatric DTC remains much rarer than adult DTC (6, 7). Studies have shown significant differences between pediatric and adult DTC in the clinical and histopathological characteristics, molecular genetics, the rates of lymph nodes and distant metastases (DM) and the outcome (8–12). Several factors are associated with the outcome of DTC, including the size of the tumor, histological subtype, gross extrathyroidal invasion and DM (13, 14). DM is a major determinant of the outcome (15). DM are relatively rare in adult DTC ranging between 3-5% but more common at higher rates of 6-40% in pediatric DTC (3, 16). Previous studies suggested that the pattern and outcome of DM are different between pediatric and adult patients with DTC (8–10, 16). However, no direct comparison between these two entities has been reported. We have recently studied the factors associated with DM in pediatric and adult patients (17, 18). We noticed differences in the pattern, extent and distribution of DM between the two age groups. In this study, we directly compared DM in pediatric and adult DTC with primary objectives to understand the differences in the characteristics of the underlying DTC, the pattern of DM, response to therapy and outcome.

## Patients and methods

This is a retrospective study of 2 cohorts of patients comprised of pediatric and adult thyroid cancer with DM managed at the same institution. The aims were to describe their clinical features, management and outcome and contrast differences between the 2 cohorts as appropriate. To achieve this, we reviewed all cases of DTC seen at King Faisal Specialist Hospital and Research Centre (KFSHRC), Riyadh, Saudi Arabia between November 2002 and December 2022. KFSHRC is the main referral tertiary care center for thyroid and other cancers in Saudi Arabia where most cases of thyroid cancer are referred to from all regions of the country. We included all patients with DM of DTC seen during this period and excluded those with DM who had no follow up data (4 pediatric and 9 adult patients). During the study period, a total of 5584 DTC patients were managed;196 (3.5%) had pediatric DTC (≤ 18 years old at the initial diagnosis) and 5388 patients (96.5%) had adult DTC (> 18 years at the initial diagnosis). Of these, 35 (17.9%) out of the 196 pediatric and 79 (1.47%) out of the 5388 adult patients developed DM. These DM were diagnosed at the time of initial thyroid surgery, either in the preoperative evaluation or within 12 weeks of surgery during the evaluation for radioactive iodine ablation in 30 pediatric and 66 adult patients. DM were diagnosed in the remaining 5 pediatric and 13 adult patients during the follow-up after the first year post radioactive iodine. We compared the initial characteristics, histopathological features, therapy and outcome between patients with DM in the two age groups. The study was approved by an Institutional Review Board approval (RAC# 2130015) and a waiver of consent from the Ethics Committee of the King Faisal Specialist Hospital & Research Centre (KFSHRC), Riyadh, Saudi Arabia.

### Statistical analysis

Continuous variables are expressed as median and ranges and analysed by t-test or Wilcoxon rank sum test. Categorical variables are expressed as rates and proportions and analysed by Chi-square and Fisher exact test. Differences in survivals were represented by Kaplan-Meier curves and compared by Log-rank test. A two-sided p value of < 0.05 was considered significant.

## Results

### Initial characteristics, management and histopathological features

The median age was 16 years (range, 5-18) and 55 years (range 21-82) and the female-to-male ratio was 3.4:1 and 1.7:1, for patients with pediatric and adult DM, respectively (**Table 1**). The number of pediatric patients with distant metastases ranges between 1–4 cases per year and there was no trend of increasing or decreasing pattern over the 20-year period. Total thyroidectomy was performed in 32/35 (91.4%) of pediatric and 74/79 (93.7%) of adult patients. Central and/or lateral lymph node dissection was also perfomed in 33/35 (94.3%) pediatric and 51/79 (64.6%) adult patients. Radioactive iodine (RAI) ablation/therapy was administered to all patients except one adult patient who refused further therapies. The majority (74.3%) of pediatric DTC were classic PTC and other subtypes of DTC were rare (**Table 1**). By contrast, high-grade subtypes were common in adult DTC including tall cell, columnar cell, oncocytic and widely invasive follicular thyroid cancer (**Table 1**). Tumor size tended to be larger in adult DTC but not significantly different from pediatric DTC while tumor multifocality, and extrathyroidal extension were more common in pediatric than adult DTC (**Table 1**). There was no difference in the rate of lympho-vascular and vascular invasion and lymph node metastases between adult and pediatric DTC (**Table 1**). Due to the presence of DM in both groups, TSH suppression (TSH < 0.1 mU/l) was maintained with no differences between the pediatric and adult groups with most readings <0.1 mU/l and occasional readings between 0.1-0.3 mU/l (normal range for our laboratory is 0.5-4.2 mU/l).

**Table 1:** Comparison of the demographic characteristics, initial management and histopathological features of 35 pediatric and 79 adult patients with DM from DTC.

Feature	Pediatric DTC (N = 35) No. (%)	Adult DTC (N = 79) No. (%)	P value
Age, median (range)	16 (5-18)	55 (21-82)	<0.0001
Female: Male	27:8 (3.4:1)	50:29 (1.7:1)	<0.0001
Initial thyroid surgery			
Total thyroidectomy	32 (91.4)	74 (93.7)	1.00
Hemithyroidectomy		3 (3.8)	
Partial thyroidectomy	2 (5.7)	2 (2.5)	
Other type of surgery		1 (1.3)	
No surgery	1 (2.9)	0	
Lymph node dissection			
No lymph node dissection (LND)	2 (5.7)	28 (35.4)	0.005
Berry picking	4 (11.4)	10 (12.7)	
Central LND	1 (2.9)	4 (5.1)	
Bilateral lateral LND	3 (8.6)	5 (6.3)	
Unilateral lateral LND		6 (7.6)	
Central +Unilateral lateral LND	2 (5.7)	13 (16.5)	
Central +Bilateral Lateral LND	21 (6)	10(12.7)	
Others	2 (5.7)	3 (3.8)	
Tumor types			
Classic papillary thyroid cancer (PTC)	26 (74.3)	26 (32.9)	<0.0001
Follicular variant PTC	3 (8.6)	14 (17.7)	
Tall cell variant PTC	0	5 (6.3)	
Diffuse sclerosing variant PTC	4 (11.4)	2 (2.5)	
Columnar variant PTC	0	5 (6.3)	
Follicular TC, minimally invasive	1 (2.9)	0	
Follicular TC, Widely invasive	0	12 (15.2)	
Oncocytic TC	0	4 (5.1)	
Poorly differentiated TC	1 (2.9)	9 (11.4)	
Others	0	2 (2.5)	
Tumor characteristics			
Size, mean ± SD (range)	4.5 ± 1.8 (1.4-9.0)	5.6 ± 2.9 (0.7-13)	0.07
Tumor multimodality	20/26 (76.9)	27/57 (47.4)	0.01
Extrathyroidal extension	26/29 (89.7)	42/62 (67.7)	0.02
Lymphovascular invasion	16/17 (94.0)	49/59 (83.0)	0.43
Vascular invasion	31/33 (92.9)	32/37 (86.5)	0.43
Lymph node metastases			
No LN removed	2 (5.7)	28 (25.4)	0.0005
No Lymph node metastases (LNM)	5 (14.3)	11 (13.9)	
Central LNM	1(2.9)	7 (8.9)	
Lateral LNM	2 (5.7)	8 (10.1)	
Lateral and central LNM	22 (62.9)	20 (25.3)	
LNM (Site unclear)	4 (11.4)	3 (3.8)	
LNM/LND	29/33 (87.9)	38/49 (77.6)	0.26
Distant metastases			
Distant metastases at diagnosis	30 (85.7)	64 (81.0)	0.60
DM during follow-up	5 (14.3)	15 (19.0)	

### Distant metastases: pattern and distribution

Pediatric DM mostly involved a single organ (34/35) and predominantly the lungs (32/35), rarely the bone (2/35) and very rarely the brain (1/35) (**Table 2**). By contrast, adult DM were frequently multi-organ (45/79) and less frequently single-organ in only 34 patients (**Table 2**).

**Table 2:** Comparison of distribution and management of pediatric and adult DM.

Feature	Pediatric DTC (No. 35) No. (%)	Adult DTC (No. 79) No. (%)
Sites of DM		
Lungs only	32 (91.4)	29 (36.7)
Bone only	2 (5.7)	5 (6.3)
Lungs, bone	0	29 (36.7)
Lungs and/or bone plus:	0	16 (20.3)
Brain	1 (2.9)	9 (11.4)
Liver	0	10 (12.7)
Kidneys	0	2 (2.5)
Adrenal glands	0	2 (2.5)
Pancreas	0	1 (1.3
Muscles and SC fat	0	3 (3.8)
Breast	0	1 (1.3)
Significant loco regional disease	0	12 (15.2)
Multiple sites	1 (2.9)	45 (57%)
Initial I-131 dose, median	114 (27-211)	156 (45-211).
Further intervention		
No intervention	6 (17.1)	1 (1.3)
Further intervention	29/35 (82.9)	78/79 (98.7)
Surgery	10 (28.6)	37 (46.8)
I-131	26 (73.3)	66 (83.5)
External bean radiotherapy	1 (2.9)	49 (62.0)
Tyrosine kinase inhibitors	0	23 (29.1)
Others	1 (2.9)	0
Number of additional treatment sessions	48 (1.66 per patient)	185 (2.37 per patient)
Cumulative I-131 dose, Median (Range)	474 (83-872)	355 (49-1300)

#### Lung DM

In adults, 72 patients (91%) had lung mets, 31 (39%) micro-DM (< 1 cm) and 41 (51.9%) macro-DM. F18-fluorodeoxyglucose positron emission/computed tomography (FDG PET-CT) scans were done in 59 patients with adult DM. It was positive in 46 (78%) and negative in 13 patients. In pediatric patients, the lung DM were visible only on radioactive whole-body scans (WBS) with normal chest CT scans in 12 cases (34.3%), on WBS and as micro-metastases on CT lungs in 17 patients (48.6%) and on WBS and CT lungs as macrometastases in 6 patients (17%). FDG PET-CT scans were done only in 4 paediatric patients and were negative in all of them. Comparison of lung DM between pediatric and adult patients is summarized in **Table 3**.

**Table 3:** Comparison of lung metastases in pediatric with adult patients with differentiated thyroid cancer.

Feature	Pediatric lung metastases (N = 32)	Adult lung metastases (N = 29)	P value
Female:male	25:7	23:6	1.0
Mean age ± SD	13.7± 3.2	51± 15.7	<0.0001
Tumor type			0.025
Classic PTC	26 (81.3)	13 (44.8)	
Follicular variant PTC	2 (6.3)	5 (17.2)	
Diffuse sclerosing variant PTC	3 (9.4)	1 (3.4)	
Poorly differentiated thyroid cancer	1 (3.1)	1 (3.4)	
Tall cell variant PTC	0	4 (13.3)	
Widely invasive FTC	0	1 (3.4)	
Oncocytic PTC	0	1 (3.4)	
Columnar cell variant PTC	0	3 (4.9)	
Treatments received after initial surgery and first dose I-131			0.034
None	6 (18.8)	0	
Additional surgeries only	1 (3.1)	0	
Additional I-131 only	12 (37.5)	13 (44.8)	
External beam radiotherapy only	0	3 (10.3)	
Tyrosine Kinase inhibitors only	0	2 (6.9)	
Multiple therapeutic interventions*	13 (40.6)	11 (37.9)	
Final outcome			
Excellent response	1 (3.1)	0	<0.0001
Indeterminate biochemical response, no structural disease*	5 (15.6)	0	
Structurally incomplete	25 (78.1)	15 (51.7)	
• Non-progressive metastases	25 (78.1)	13(44.8)	
• Progressive metastases	0	2 (6.9)	
Death due to DTC	1 (3.1)	9 (31.0)	0.004
Death due to another cause	0	0	
Lost for follow up	0	5 (17.2)	
Duration in months (IQ Range)	120 (62.7-192.25)	108 (42-144)	0.29

*These include two or more of the following: additional surgeries, I-131, external beam radiotherapy or tyrosine kinase inhibitors.

#### Bone DM

Bone DM developed in 50 adult patients (63.3%). The sites included spine alone in 3 patients, ribs in 1 patient, extremities in 3 patients and skull alone in 1 patient. The other 42 patients (84% of those with bone DM) had bone DM in multiple sites. Only 2 pediatric patients (5.7%) had isolated bone DM and 1 (2.9%) had bone DM along with lung macro-DM and brain micro-DM. The first patient with isolated bone DM was a 12-year-old-girl who had FTC with extensive bone but no lung metastases. She was treated with one dose of I-131 (119 mCi). She is now 20-year old and in excellent response (negative I-123 WBS, ultrasound of the neck, FDG PET-CT WBS, undetectable thyroglobulin (Tg) and negative anti Tg Abs). The second patient with isolated bone metastases had 3 RAI therapies and remained stable without further progression. I-123 WBS became negative, Tg was undetectable but this patient had Tg mutation that led to undetectable Tg and was described before (19). CT scans and MRI showed sclerotic bone DM and FDG PET-CT was negative. The third patient is described below under other common sites

### Other uncommon sites

Only one pediatric patient had multi-organ DM. This was a 13-year-old girl who presented with extensive lung and bone DM and later developed brain micro-metastases (<1 cm). This patient was positive for *RET* fusion and was treated with selpercatinib with good response for 4 years but her DM recently started to lose sensitivity and are now slowly progressive although she remains alive and stable. None of the pediatric patients had DM in unusual sites. By contrast, adult DM were frequently multi-organ (45/79) involving isolated or multiple organs as follows: lungs (72/79), bone (50/79), brain (9/79), liver (10/79), kidneys (3/79), adrenal glands (2/79), breast (1/79) and soft tissues and muscles (3/79) (**Table 2**).

### Management

Following the initial therapy (neck surgery and first dose RAI), 28 pediatric patients needed 48 additional therapy sessions [surgeries, RAI, external radiotherapy, and/or tyrosine kinase inhibitors (TKI)] while all except one adult patient received 185 additional therapy sessions (2.37 per patient). The details of additional therapies are summarized in **Table 2**.

In adults, one patient did not have any additional therapy as she refused further therapies. The other 78 patients (98.7%) received a total of 185 therapeutic sessions with one or more additional therapies for DM per patient (**Table 2**). In pediatric patients, after the initial management (thyroid surgery and I-131), 7 patients ((20%) did not receive any further therapy. Twenty-eight patients (80%) received 1 or more therapies (**Table 2**).

### Final outcome

**Figure 1:**
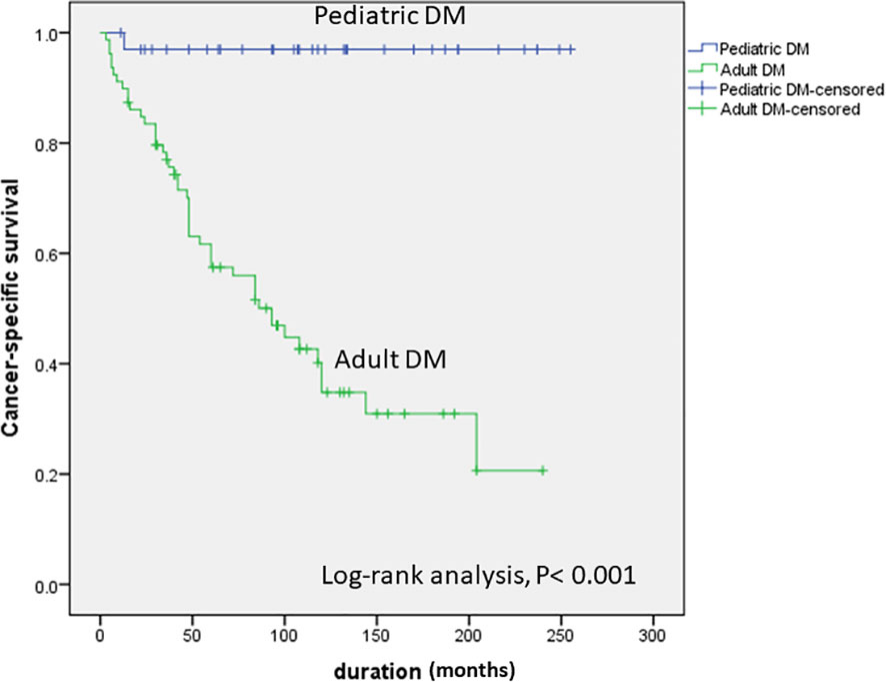
Kaplan-Meier curve showing a significant difference in the cancer-specific survival between pediatric and adult DTC with distant metastases.

Over a median follow-up of 116 months (62.5-188.75) for pediatric and 65 (34-108) for adult patients, 3 (8.6%) vs. 1 (1.3%) achieved the status of no evidence of disease (NED), while 1 (2.9%) vs. 6 patients (7.6%) had progressive DM and 1 (2.9%) vs. 46 (58.2%) died due to progressive DTC in pediatric and adult DM, respectively (**Table 4**). Other patients were either in an indeterminate status (5 in pediatric and zero in adult patients) or stable non-progressive metastases (25 in pediatric and 18 in adult patients) (**Table 4**). An indeterminate status was defined as presence of non-specfic radiological changes or mildly detectable Tg (suppressed level of detectable to 0.2 ng/dl or stimulated Tg between 1–10 ng/dl or presence of anti Tg antibodies). Non-progressive metastases were defined as presence of clear metastases on radiological evaluation that remains stable on repeated radiological evaluations over time. The Kaplan-Meier curve shows a very significant difference between pediatric and adult DM with only 1 cancer-related death in the pediatric and 46 cancer-related deaths in the adult DM group (**Figure 1**).

**Table 4:** Final outcome of 35 pediatric and 79 adult patients with distant metastases.

Final outcome	Pediatric DM (No. 35) No. (%)	Adult DM (No. 79) No. (%)
Excellent response	3 (8.6)	1 (1.3)
Indeterminate biochemical response, no structural disease*	5 (14.3)	0
Structurally incomplete	26 (74.3)	24 (30.4)
• Non-progressive metastases	25 (71.4)	18 (22.8)
• Progressive metastases	1 (2.9)	6 (7.6)
Death due to DTC	1 (2.9)	46 (58.2)
Death due to another cause	0	1 (1.3)
Lost for follow up	0	7 (8.9)
Duration in months (Range)	116 (62.5-188.7)	65 (34-108)

*Radiological evaluation was negative but Tg was in the indeterminate range (suppressed 0.2-<1 ng/dl, stimulated 1–10 ng/dl).

## Discussion

In this study, we described a cohort of pediatric patients with DTC DM and another cohort of adult patients with DTC DM and compared their clinical, pathological and radiological features and management and outcome. The patients were consecutive patients seen over 20 years and managed at the main referral center in Saudi Arabia by experienced endocrinologists in thyroid cancer management working in the same department and following the same protocol at the time of patients’ presentation. This decreases bias arising from different management practices and makes the comparison likely relevant. The differences were striking in terms of the pattern, distribution, response to therapy and outcome. The hallmark of these differences is that adult DM, compared to pediatric DM, frequently involved multiple organs including soft tissues and unusual sites, showed more resistance to therapy, and were commonly associated with the progression of the disease and very high mortality. On the other hand, pediatric DM predominantly involved single organs, and associated with good response to therapy and stability. Mortality was exceptionally low occurring only in one of the whole 35-patient cohort.

These differences might be due to differences in the underlying molecular genetics of DTC in the two age groups. Previous studies have shown that the *BRAF*^V600E^ mutations are about 50% less common in pediatric compared to adult DTC and *TERT* promoter mutations are exceedingly rare (12, 20–25). These mutations have been shown repeatedly to be associated with more aggressive features of DTC and higher recurrence and mortality, especially when they occur together in the same tumor (26–31). Therefore, it is likely that the underlying molecular genetics played an important role in these differences between pediatric and adult DM in the current study. One potential implication of the low rate of *BRAF*^V600E^ and *TERT* promoter mutations is the increased avidity and responsiveness to I-131 (32) which is the primary modality of therapy for DM in DTC. Another observation in our cohorts is that the adult DM were characterized by a relatively high number of aggressive DTC variants and poorly DTC. These variants are more invasive and less responsive to different modalities of therapy including I-131 and tyrosine kinase inhibitors (33, 34). Another feature of the adult DMs in this study is the high rates of bone and unusual sites of DM such as the brain and liver. Previous studies have shown less favorable outcomes of DM in these sites (35–37).

In a recent large study of 2411 adult patients with DM registered in the SEER database during the period 2010-2019, PTC of all subtypes was the most common type (86%) followed by FTC (10.6%) and oncocytic (3.4%) types. DM were most common in the lungs (33.7%), followed by bone (18.9%), brain (2.3%) and liver (2.3%). Mortality was 23.1%, lower than the mortality in adult DM in the current study but still substantial. Age, tumor size > 4 cm, and lung metastases were associated with increased mortality (35). Another previous study that has also used the SEER database between 1988 and 2009, compared DTC patients with DM at presentation (1291 patients) with patients without DM at presentation (58,518 patients) patients. Male, gender, large tumor size > 4 cm, not having surgery or I-131, lymph node metastases, single status, black or other ethnic status, and follicular and oncocytic subtypes were all associated with increased mortality. Cancer-specific mortality was higher in patients with DM and has not improved over the study time. In another study that we published recently and included most patients included in this study, we analyzed the factors associated with mortality in adult patients with DM, we found age ≥ 50 years and the presence of bone DM to be significantly associated with cancer-specific mortality (38). Unlike the previous study, lung metastases were not associated with cancer-specific mortality, probably because of their common occurrence in most patients who died and in those who were still surviving at the time of the study (38).

In pediatric thyroid cancer, Liu Z. et al. studied factors associated with DM in 1376 pediatric thyroid cancers (age 2-18 years) from the SEER database registered between 2003 and 2014. Of the total cohort, 52 (3.8%) had DM and 1324 (96.4%) did not. Factors associated with the development of DM were age at diagnosis, T-stage and N-stage (TNM staging system) (39). In that study, only 3 patients died from thyroid cancer (39). In another study from Japan, of 171 pediatric patients (<19 years old) seen between 1979 and 2014, 29 had DM, all in the lungs (40). Similar to our study, the pattern was macro-metastases (4 patients), micro-metastases (19 patients), or DM seen only on RAI WBS (4 patients). Excellent responses were observed more frequently in the last group. Factors associated with DM-free survival were female sex, clinical LN metastases, extrathyroidal extension and number of LN metastases. Using these factors, they classified their patients into low risk (no risk factor), intermediate risk (1 risk factor) and high risk (≥ 2 risk factors). The 20-year-DM-free survival was 99%, 71.7% and 28.9%, respectively. However, only 3 deaths occurred over the long follow-up period (40). Hay I. et al. reported the outcome of 215 pediatric thyroid cancer (< 21 years of age at the time of diagnosis) managed over 74 years at the Mayo Clinic (41). Only 2 patients had disease-specific mortality but there was an excess of mortality related to non-thyroid malignancy (15 of 22 deaths) raising suspicion about the contribution of RAI to the development of these malignancies as 73% of them received RAI during childhood for DTC (41). A cohort of 148 pediatric patients with DM managed over 74 years (1946-2019) at MD Anderson Cancer Center was also reported (42). Although 93% continued to have persistent disease at the last evaluation, only 8 patients (5%) died due to DTC and the median overall and median disease-specific survival were 50.7 years and 52.8 years, respectively. In 64 of 69 tumor samples evaluated for molecular alterations, RET::PTC fusions were present in 59% and *NTRK* fusions in 28%. *BRAF*^V600E^ was present only in 13%. (42).

The strength of this study is that it included all patients seen in one institution and managed by the same team over a 20-year study period. However, it has weaknesses including the retrospective non-controlled nature of the study of a single center limiting its generalizability and the absence of molecular testing. Although the number of patients with DM is relatively large, it remains small for inferential statistical analysis. Although this study shows clear differences in the pattern and outcome of pediatric vs. adult DMs, these remains descriptive and not comparative. Although pediatric thyroid cancer is more likely to be associated with DM, the differences in the incidence of DM between pediatric and adult patients in this study is striking (17.9% vs. 1.47%) raising questions about potential selection bias. However, the differences in the underlying tumor types, DM pattern and distribution, response to therapy and the course of the disease are clear. The mortality rates are strikingly different with a median survival much longer for pediatric than adult patients with DM.

## Data Availability

The original contributions presented in the study are included in the article/supplementary material. Further inquiries can be directed to the corresponding author.
